# Development and validation of a random forest algorithm for source attribution of animal and human *Salmonella* Typhimurium and monophasic variants of *S.* Typhimurium isolates in England and Wales utilising whole genome sequencing data

**DOI:** 10.3389/fmicb.2023.1254860

**Published:** 2024-03-12

**Authors:** Jaromir Guzinski, Yue Tang, Marie Anne Chattaway, Timothy J. Dallman, Liljana Petrovska

**Affiliations:** ^1^Animal and Plant Health Agency, Bacteriology Department, Addlestone, United Kingdom; ^2^Gastrointestinal Bacteria Reference Unit, UK Health Security Agency, London, United Kingdom

**Keywords:** source attribution, *Salmonella* Typhimurium, machine learning, random forest, core-genome multi locus sequence typing, bacterial genomics

## Abstract

Source attribution has traditionally involved combining epidemiological data with different pathogen characterisation methods, including 7-gene multi locus sequence typing (MLST) or serotyping, however, these approaches have limited resolution. In contrast, whole genome sequencing data provide an overview of the whole genome that can be used by attribution algorithms. Here, we applied a random forest (RF) algorithm to predict the primary sources of human clinical *Salmonella* Typhimurium (*S.* Typhimurium) and monophasic variants (monophasic *S.* Typhimurium) isolates. To this end, we utilised single nucleotide polymorphism diversity in the core genome MLST alleles obtained from 1,061 laboratory-confirmed human and animal *S.* Typhimurium and monophasic *S.* Typhimurium isolates as inputs into a RF model. The algorithm was used for supervised learning to classify 399 animal *S.* Typhimurium and monophasic *S.* Typhimurium isolates into one of eight distinct primary source classes comprising common livestock and pet animal species: cattle, pigs, sheep, other mammals (pets: mostly dogs and horses), broilers, layers, turkeys, and game birds (pheasants, quail, and pigeons). When applied to the training set animal isolates, model accuracy was 0.929 and kappa 0.905, whereas for the test set animal isolates, for which the primary source class information was withheld from the model, the accuracy was 0.779 and kappa 0.700. Subsequently, the model was applied to assign 662 human clinical cases to the eight primary source classes. In the dataset, 60/399 (15.0%) of the animal and 141/662 (21.3%) of the human isolates were associated with a known outbreak of *S.* Typhimurium definitive type (DT) 104. All but two of the 141 DT104 outbreak linked human isolates were correctly attributed by the model to the primary source classes identified as the origin of the DT104 outbreak. A model that was run without the clonal DT104 animal isolates produced largely congruent outputs (training set accuracy 0.989 and kappa 0.985; test set accuracy 0.781 and kappa 0.663). Overall, our results show that RF offers considerable promise as a suitable methodology for epidemiological tracking and source attribution for foodborne pathogens.

## 1 Introduction

Salmonellosis, one of the most common food-borne illnesses in both, the developed and developing countries (Majowicz et al., 2010; Scallan et al., 2011; Pires et al., 2021), is a disease that is associated with diarrhoea, fever and abdominal pains that occasionally can lead to death (Fabrega and Vila, 2013; Andino and Hanning, 2015). Salmonellosis was the second most reported zoonotic disease in the EU in 2020 [European Food Safety Authority (EFSA) and European Centre for Disease Prevention and Control (ECDC), 2021] and second most reported bacterial enteric disease in the US in 2022 [Centers for Disease Control and Prevention (CDC), 2023]. The annual costs associated with salmonellosis in 2010 in the US were estimated to be in excess of 2.5 billion USD for 1.4 million cases (Scallan et al., 2011; Andino and Hanning, 2015).

Current classification divides the genus *Salmonella* into two species: *enterica* and *bongori*. *Salmonella enterica* is further divided into six well defined subspecies that comprise over 2,600 distinct serovars (Issenhuth-Jeanjean et al., 2014). *Salmonella enterica* subspecies *enterica* (I) is responsible for the majority of *Salmonella* infections in warm blooded animals (Porwollik et al., 2004), although *S. Enterica* subspecies *diarizonae* (IIIb) serovar 61:k:1,5,(7) is host adapted and endemic in Sheep in multiple countries (Davies et al., 2001; Alvseike et al., 2004; Sörén et al., 2015; Methner and Moog, 2018) and *S. Enterica* subspecies *arizonae* (IIIa) can infect avian and mammalian host species (Katribe et al., 2009). The majority of human salmonellosis cases are caused by a minority of the described *Salmonella* serovars. For example, in the US in 2016 just 20 serovars were reported as a cause of >80% of human infections with over one-third of cases due to just three serovars: *S.* Typhimurium, *S.* Enteritidis, and *S*. Newport [Centers for Disease Control and Prevention (CDC), 2018]. Similarly, in the United Kingdom (UK), *S.* Typhimurium and *S.* Enteritidis were responsible for approximately 50% of non-typhoidal *Salmonella* infections in England in 2019 (UKHSA, 2021). Worldwide, World Health Organization (WHO) data reported that *S.* Enteritidis and *S.* Typhimurium are the two serovars most frequently isolated in clinical practice (Fabrega and Vila, 2013).

The main cause of human non-typhoidal salmonellosis is the ingestion of contaminated food, or, especially in low to middle income countries, contaminated water (Fabrega and Vila, 2013). The source of such contamination is typically *Salmonella* in faeces of an infected primary animal host (or, more rarely, human host) contaminating the water supply or plant based foodstuffs, or food products obtained from an infected primary animal host, including meat (typically pork, beef, poultry, or mutton/lamb), eggs, or diary (Hald, 2013). Cross-contamination at the different stages of the food production chain (e.g., at an abattoir or a food processing plant) can also be a significant cause of contamination of foodstuffs and hence human salmonellosis infection (Andino and Hanning, 2015). Additionally, *S.* Typhimurium and monophasic *S.* Typhimurium have been shown to persist in farm environments for extended periods of time and have also been isolated from animal feed and feed ingredients (Andino and Hanning, 2015; Gosling et al., 2018; Harrison et al., 2022). *Salmonella* Typhimurium and monophasic variants of *S.* Typhimurium can infect a wide range of animal species, of which the most relevant primary sources in terms of the potential for human infection are various livestock animals and poultry, companion animals and pets (horses, dogs, and cats), and wild game mammals and birds. Whether the primary host displays any symptoms of infection is dependent on the host species and the *Salmonella* serovar. Primary host can often act as a reservoir of infection where the bacterium lives and multiplies in the large intestine and associated lymphoid tissue. Given the diverse range of potential primary animal hosts, and thus the numerous and complicated transmission pathways of these zoonotic pathogens (Hald, 2013), it can be difficult to determine the primary source of the *S.* Typhimurium and monophasic *S.* Typhimurium human infections for both sporadic cases and outbreaks. This information is critical for formulating efficient strategies for mitigating *S.* Typhimurium and monophasic *S.* Typhimurium infection spread in the human population. Hence, development of attribution methodologies to better understand pathogen transmission to humans is crucial.

Historically, source attribution efforts have relied on frequency-matching models [e.g., the Dutch and Hald (“Danish”) models], which rely on the one-to-one matching of microbial subtypes, defined either by phenotyping (e.g., serotyping) or genotyping (e.g., 7-gene MLST), in humans and potential sources, or on probabilistic population genetics approaches that utilize genetic markers derived from genotypic subtyping methods. These methodologies have been reviewed in several recent publications (e.g., Pires et al., 2009, 2014; Mughini-Gras et al., 2018, 2019). Pires et al. (2014) reviewed the utility of these approaches for attribution of human salmonellosis cases. The high-throughput sequencing of bacterial strains has been increasingly used for routine surveillance and outbreak investigations. Generated whole genome sequencing (WGS) data can additionally be of use to accurately discriminate between human infecting pathogens originating from different primary sources thus allowing for development and application of ever more sophisticated attribution models (Franz et al., 2016).

Machine learning (ML) models are computer algorithms that improve with experience and have been increasingly applied to analyse various large and complex genetic and genomic datasets (Libbrecht and Noble, 2015). Recently, there has been a proliferation of studies applying ML algorithms to WGS data of zoonotic bacterial isolates to answer questions related to attribution [e.g., primary host species of *S.* Typhimurium (Zhang et al., 2019, Munck et al., 2020a), food source of *Listeria monocytogenes* (Tanui et al., 2022), geographic origin of *S.* Enteritidis (Bayliss et al., 2023)], disease risk in humans (Njage et al., 2019a), or host disease severity (Karanth et al., 2022). RF models are widely used supervised classification ML algorithms that have been applied in a range of different research fields and are particularly useful for making predictions based on the WGS data (Ogutu et al., 2011). A RF algorithm generates multiple decision trees and subsequently aggregates the output produced by each individual decision tree to arrive at the consensus set of predictions. Importantly, the different decision trees are uncorrelated as each tree is exposed to a random subset of the data (variables and model features), which minimizes bias and error. Using this approach, here we describe an application of a supervised classification RF algorithm on a WGS derived set of core genome MLST (cgMLST) genetic markers to assign the primary sources to 662 *S.* Typhimurium and monophasic *S.* Typhimurium sporadic and outbreak human clinical cases detected between 2012 and 2018 in England and Wales.

## 2 Materials and methods

### 2.1 Strains and sequencing

Prior to sequence quality control (QC), the animal isolate dataset comprised the WGS data of 463 *S.* Typhimurium and monophasic *S.* Typhimurium sequence type (ST)19, ST34, ST128, ST313, ST323, ST568, and ST2105 isolates. All STs belonged to eBurst group (eBG) 1, with the exception of ST2105 that belonged to eBG167. The analysed animal isolates were collected by the Animal and Plant Health Agency (APHA) between 2012 and 2020 (majority of these isolates were from 2013–2018) as part of routine surveillance of livestock farms across England and Wales, monitoring, control programs, outbreak investigations, and for research projects. The isolates originated from eight primary source classes (animal species or groups of animal species). Grouping of primary hosts into the distinct primary sources was performed as described in Munck et al. (2020b) for the UK animal isolates: Cattle, Pigs, Sheep, OtherMammals (companion animals that were mostly dogs and horses), Broilers, Layers (egg laying hens), Turkey, Game (game birds: pheasant, quail, pigeon).

The pre-QC human isolate dataset comprised the WGS data of 852 *S.* Typhimurium and monophasic *S.* Typhimurium ST19, ST34, ST213, ST313, ST323, and ST3235 (all eBG1) isolates collected from salmonellosis patients in England and Wales between 2012 and 2018. Only a few human isolates in this dataset were from prior to 2014. The WGS data and the metadata of the human isolates were provided by the United Kingdom Health Security Agency (UKHSA).

Animal isolates were sequenced at APHA Weybridge using either the MiSeq or NextSeq benchtop Illumina sequencers. Paired-end libraries were prepared with the Illumina Nextera XT DNA Library Preparation Kit from DNA extracted with the MagMAX CORE Nucleic Acid Purification Kit (ThermoFisher Scientific, Applied Biosystems, Foster City, CA) following the manufacturer’s instructions. Human isolates were sequenced at UKHSA as previously described (Chattaway et al., 2019). The fastq files of the 852 human isolates were downloaded from the NCBI GenBank Sequence Read Archive (BioProject PRJNA248792) using fasterq-dump of SRA Toolkit v2.9.6^1^. Both the animal and human isolate datasets included samples that were linked with the 2015–2018 *S.* Typhimurium DT104 outbreak in England and Wales [Animal and Plant Health Agency (APHA), 2017].

### 2.2 Quality control of the WGS data

The whole genome sequences of the 1,315 animal and human *S.* Typhimurium and monophasic *S.* Typhimurium isolates were subjected to rigorous filtering prior to usage in the downstream analyses. BBDuk software (Bushnell, 2014) was used for removing adapter sequence and terminal bases with PHRED scores below 20 from each of the reads. Trimmed reads below 50 bases were filtered out. If just one of a pair of reads was under 50 bases, the other read in the pair was also removed. FastQC (Andrews, 2010) was run on the WGS data before and after read trimming to assess improvements in sequence quality. *De novo* genome assemblies were generated from the trimmed fastq reads using shovill v0.9.0^2^ and analysed with QualiMap 2 (Okonechnikov et al., 2016) and Quast v5.0.2 (Gurevich et al., 2013) to obtain the mean coverage across the genome and evaluate the quality metrics (based on contigs of size 500 bases or larger). Only isolates with mean depth of sequence data post read filtering of at least 30X, genome assembly size between 4,750,000 and 5,250,000 bases, N50 >30,000, and the number of assembled contigs <500, were retained for cgMLST allele calling. The final dataset comprised 1,244 isolates, of which 435 were animal and 809 human isolates.

### 2.3 Scoring of cgMLST alleles

MentaLiST (Feijao et al., 2018) was used to call the cgMLST alleles against the 3,002 locus cgMLST EnteroBase scheme (version from September 2019) (Alikhan et al., 2018; Zhou et al., 2020) from the trimmed R1 and R2 fastq files of the 1,244 retained isolates. Default MentaLiST parameters were used but the minimum kmer depth required to call an allele was set to five. Novel alleles detected after the first MentaLiST run were introduced into the cgMLST scheme following the steps outlined in the MentaLiST manual. The −*t* parameter was set to one, and the –*m* parameter was set to 10. Second MentaLiST run with the updated cgMLST scheme produced several novel alleles that were generated from the novel alleles introduced after the first MentaLiST run at three different cgMLST loci in 10 different isolates. Novel alleles identified after the second MentaLiST run were treated as missing data.

Where there was an indication of multiple possible alleles (i.e., more than one allele with 100% kmer coverage), the allele calls with the highest number of votes were accepted and included in the downstream analyses. The cgMLST alleles with kmer coverage below 100% of the minimum kmer depth required to call an allele were treated as missing data. If an isolate had missing data at greater than 5% of the 3,002 cgMLST loci, it was not included in the subsequent analyses. Using these criteria, a further 183 isolates were removed from the dataset. The 1,061 retained isolates comprised 399 (ST19, ST34, ST128, ST313, ST323, ST568—all eBG1) animal and 662 (ST19, ST34, ST213, ST313, ST323—all eBG1) human *S.* Typhimurium and monophasic *S.* Typhimurium isolates (Supplementary Table S1). Missing data (1.1% of all allele calls) within the retained dataset was imputed utilising an iterative imputation method based on a random forest implemented in the missForest R package (Stekhoven and Bühlmann, 2012). Imputation was performed on all 1,061 isolates with the default parameters.

### 2.4 Phylogenetic tree construction and hierarchical clustering of 399 animal isolates

Phylogenetic analyses were carried out to investigate clustering according to primary source of the 399 animal isolates originating from eight primary source classes: 77 isolates from Cattle, 165 from Pigs, 47 from Sheep, 56 from OtherMammals (including 1 from ferret, 4 from cats, 20 from dogs, 31 from horses), 19 from Broilers, 7 from Layers, 11 from Turkey, and 17 from Game (including 4 from pigeon, 5 from pheasant, 8 from quail) (Supplementary Table S1).

According to the metadata recorded in the APHA LIMS database, the host type of the 399 animal isolates was an animal species as specified above. However, inspecting the farm sampling sheets indicated that 285 isolates were sampled from an actual animal (including animal post-mortem samples but also faeces sampled from a pen floor or poultry house boot swab samples) and 27 isolates were sampled from the farm environment (including samples from mud puddle, farm equipment, dust). For 87 isolates there was no data on whether these isolates were sampled from animal hosts or farm environment although many of the postcodes where these samples were obtained from indicated these isolates were sampled from a livestock farm (Supplementary Table S1). Six of the unspecified source isolates were collected from farms from which one or more of the actual animal source isolates were also collected from. For the 46 unspecified source OtherMammals isolates the sampling locality situation was somewhat different as many of the sampling postcodes that these isolates were obtained from indicated individual pet owner or veterinary surgery addresses but given this class of hosts (i.e., pet animals such as dogs or horses) it is highly plausible that all those isolates were sampled from individual pets.

A multiple sequence alignment (MSA) was computed with snippy v 4.4.5^3^ from the trimmed WGS data of 399 animal isolates and the outgroup strain *S.* Typhimurium eBG138, ST36, SRR8820637 against the reference strain *S.* Typhimurium eBG1, ST19, LT2 AE006468. Recombination events were removed using Gubbins (Croucher et al., 2015), and polymorphic sites were extracted from the filtered MSA with SNP-sites (Page et al., 2016). RAxML-NG v0.9.0 (Kozlov et al., 2019) was used for phylogeny construction based on the resulting core single nucleotide polymorphism (SNP) alignment, which comprised 5,683 sites. RAxML-NG was run with the generalized time-reversible (GTR) nucleotide substitution model plus gamma correction, searching 100 trees (50 random and 50 parsimony-based starting trees) to find a tree with the best scoring topology. Branch support was computed via 2,500 bootstrap replicates (Felsenstein’s bootstrap proportions). The Newick file of the best scoring maximum likelihood tree with the bootstrap support values was imported into iTol (Letunic and Bork, 2019) for tree display and annotation. The tree was rooted at the SRR8820637 outgroup strain.

A Bayesian clustering algorithm (BAPS) that inferred the population genetic structure of the 399 animal isolates was implemented through the rhierbaps R package (Cheng et al., 2013; Tonkin-Hill et al., 2018). The program was run on the SNP alignment after removal of the reference and the outgroup strains. The resulting alignment comprised 5,336 polymorphic sites. Clustering was performed with three hierarchical levels and 40 initial clusters. The n.extra.rounds parameter was set to 100,000,000 to ensure convergence of the algorithm.

### 2.5 SNP address

SNP address strain level nomenclature (Dallman et al., 2018) was employed to assign a SNP address to the each of the 1,061 retained isolates using SnapperDB. The SNP address 60.11.15.16.458.459.x was used to define the 201 DT104 outbreak related isolates (eBG1, ST19) at the 5 SNP threshold.

A genetic relationship amongst the 60 animal (26 from Cattle, 1 from Pigs, 20 from Sheep, and 13 from OtherMammals) and 141 human DT104 outbreak related isolates was explored by computing a phylogenetic tree following the steps outlined above. The core SNP alignment for this dataset comprised 868 variable sites.

### 2.6 Feature selection (data pre-processing and feature selection algorithms)

Feature selection was performed on the 3,002 cgMLST loci prior to their utilization as the predictors or model features to minimize the redundancy, and hence model running time, and to avoid overfitting. Feature elimination was performed on the 399 animal isolate dataset in several steps, by progressively reducing the number of cgMLST loci to retain only those that exhibited allele calls that were the most useful for distinguishing between isolates from the different primary source classes. First, the caret R package (Kuhn, 2008) was used to eliminate 835 zero variance loci (loci monomorphic within the 399 isolates) and 333 near zero variance loci (loci with two alleles only, one allele appearing in 398 out of 399 isolates). Next, the remaining 1,834 loci were checked for correlation with the *findCorrelation* function of caret. Nine hundred and thirty two loci with an absolute correlation value of minimum 0.9 were eliminated and 902 loci with an absolute correlation below 0.9 were retained.

The 902 remaining cgMLST loci were subjected to two different feature selection algorithms: the *rfe* function of caret and the *Boruta* function of the Boruta R package (Kursa and Rudnicki, 2010), with the set of loci retained as model inputs based on the combined outputs of both algorithms. Prior to running the feature selection algorithms, the 399 animal isolate dataset was split in a randomised manner into the model training and test sets, with 80% of the isolates used as the training set and 20% as the test set. The 80:20 split ratio was also maintained for each of the eight primary source classes. Hence, the training set comprised a total of 322 animal isolates: 62 Cattle, 132 Pigs, 38 Sheep, 45 OtherMammals, 16 Broilers, 6 Layers, 9 Turkey, and 14 Game isolates; whereas the test set comprised a total of 77 animal isolates of which 15 were Cattle, 33 Pigs, 9 Sheep, 11 OtherMammals, 3 Broilers, 1 Layers, 2 Turkey, and 3 Game isolates (Table 1). Details of running the *rfe* (backwards feature selection) and *Boruta* (top-down feature selection) algorithms are provided in the Supplementary material. The final set of retained features comprised 130 *rfe* selected cgMLST loci plus additional 33 *Boruta* selected cgMLST loci for a total set of 163 cgMLST loci used as model features.

**Table 1:** Description of the machine learning model datasets.

Model dataset	Details	Description	Dataset function
Animal isolate training set (RF1, RF2, RF3)	322 isolates from 8 primary source classes—Cattle: 62 (19.3%), OtherMammals: 45 (14.0%), Pigs: 132 (41.0%), Sheep: 38 (11.8%), Broilers: 16 (5.0%), Layers: 6 (1.9%), Turkey: 9 (2.8%), Game: 14 (4.3%)	Primary source known and provided to the model	Used for feature selection and model training
Animal isolate test set (RF1, RF2, RF3)	77 isolates from 8 primary source classes—Cattle: 15 (19.5%), OtherMammals: 11 (14.3%), Pigs: 33 (42.9%), Sheep: 9 (11.7%), Broilers: 3 (3.9%), Layers: 1 (1.3%), Turkey: 2 (2.6%), Game: 3 (3.9%)	Primary source known but withheld from the model	Used to verify model’s ability to correctly recognize isolates originating from different primary source classes
Animal isolate training set (RF1—no DT104)	275 isolates from 8 primary source classes—Cattle: 41 (14.9%), OtherMammals: 35 (12.7%), Pigs: 132 (48.0%), Sheep: 22 (8.0%), Broilers: 16 (5.8%), Layers: 6 (2.2%), Turkey: 9 (3.3%), Game: 14 (5.1%)	Primary source known and provided to the model	Used for feature selection and model training
Animal isolate test set (RF1—no DT104)	64 isolates from 8 primary source classes—Cattle: 10 (15.6%), OtherMammals: 8 (12.5%), Pigs: 32 (50.0%), Sheep: 5 (7.8%), Broilers: 3 (4.7%), Layers: 1 (1.6%), Turkey: 2 (3.1%), Game: 3 (4.7%)	Primary source known but withheld from the model	Used to verify model’s ability to correctly recognize isolates originating from different primary source classes
Human isolates	662 human isolates	Primary source not known	Isolates assigned by the model to each of the eight primary source classes

Phylogenetic trees were constructed for the 322 training and 77 test set animal isolates utilising only the variable sites from the 163 cgMLST loci retained as ML model input. The chimeric reference genome for both trees was constructed by concatenating the sequences of allele “1,” as per the September 2019 version of the *Salmonella* cgMLST EnteroBase scheme, for each of the 163 retained cgMLST loci. Computation of the MSA, the core SNP alignment, and the phylogenetic trees was performed as described above. The training set core SNP alignment had 678 sites, and the test set core SNP alignment had 449 sites. For both trees, branch support was computed via 10,000 bootstrap replicates.

### 2.7 Random forest models applied to the full dataset

Three RF models differentiated by the model tuning procedure: RF1 and RF2 (both ran in randomForest R package; Liaw and Wiener, 2002) and RF3 (ran in ranger R package; Wright and Ziegler, 2017) (Table 2) were applied to predict the primary source classes of the training and test set animal isolates (Table 1). Subsequently, the outputs of the three models were analysed and compared. Selection between the RF1, RF2, and RF3 models was performed by comparing the accuracy (percentage of correctly classified isolates) and kappa (a measure similar to accuracy that also takes into account the possibility of the correct classification occurring by chance) of the training and test set predictions obtained for each tuned model (Table 2), and also by investigating the incorrectly assigned isolates (such as which primary source class an isolate was incorrectly assigned to). The selected model (see *Results*) was then applied to predict the primary source for each of the 662 human isolates (Table 1). The source with the highest probability of assignment was considered the model predicted source for each human isolate. For each primary source class, the sum of probabilities of assignment indicated the number of human isolates that were assigned to a source (Munck et al., 2020a). One hundred and forty one human isolates related to the DT104 outbreak were used to validate model performance by contrasting the model predicted primary sources against the epidemiologically linked primary sources. The caret R package was used for all modelling work (details in the Supplementary material).

**Table 2:** Comparison of the parameters (random forest algorithm, resampling methodology, model hyperparameters, number of hyperparameter combinations tested) and the outputs (optimal model accuracy and hyperparameters, training set accuracy, training set kappa, test set accuracy, test set kappa) for three models RF1, RF2, RF3 performed on the full animal isolate dataset and the RF1—no DT104 model performed on a dataset without the clonal DT104 animal isolates.

Model	Random forest algorithm	Resampling methodology	Model hyperparameters	Number of hyperparameter combinations tested	Optimal model accuracy and hyperparameters	Training set accuracy	Training set kappa	Test set accuracy	Test set kappa
RF1	RandomForest	Out of bag sampling (10,000 iterations)	*mtry* (1 to 163 in increments of 1)	163	0.786 (*mtry* = 109)	0.929 (95% CI: 0.895–0.954)	0.905	0.779 (95% CI: 0.670–0.866)	0.700
RF2	RandomForest	10-times repeated 10-fold cross-validation	*mtry* (1 to 163 in increments of 1)	163	0.775 (*mtry* = 49)	0.901 (95% CI: 0.863–0.931)	0.867	0.805 (95% CI: 0.699–0.887)	0.727
RF3	Ranger	10-times repeated 10-fold cross-validation	*mtry* (2 to 162 in increments of 2), *splitrule* (gini or extratrees), *min.node.size* (1, 5 to 30 in increments of 5)	1,134	0.778 (*mtry* = 40, *splitrule* = gini, *min.node.size* = 1)	0.913 (95% CI: 0.877–0.941)	0.884	0.805 (95% CI: 0.699–0.887)	0.727
RF1—no DT104	RandomForest	Out of bag sampling (10,000 iterations)	*mtry* (1 to 421 in increments of 1)	421	0.818 (*mtry* = 82)	0.989 (95% CI: 0.969–0.998)	0.985	0.781 (95% CI: 0.660–0.875)	0.663

### 2.8 Random forest model without the clonal DT104 animal isolates

To assess the influence of the clonal DT104 animal isolates on model performance, a RF1—no DT104 model was run without the 60 DT104 outbreak related animal isolates (Table 1). The RF1—no DT104 model utilized the same random forest algorithm and resampling methodology as the RF1 model (Table 2). The RF1—no DT104 model used 421 cgMLST loci as model features that were retained after applying the *rfe* and *Boruta* feature selection algorithms as described above. After model training and tuning, the RF1—no DT104 model (Table 2) was subsequently applied to predict the primary sources of the 662 human isolates.

## 3 Results

### 3.1 Phylogenetic relationship of the 399 animal isolates

**Figure 1:**
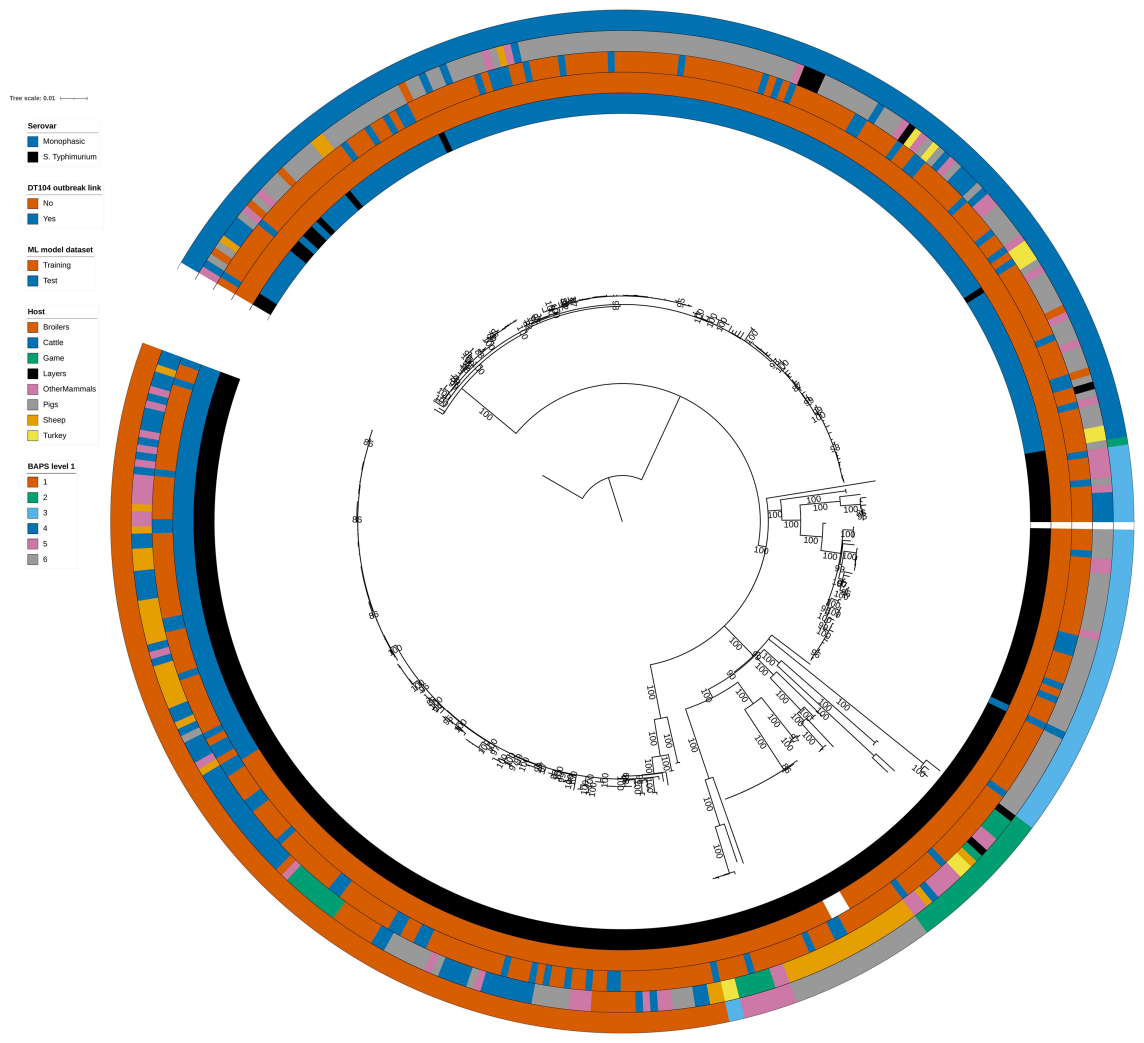
Phylogenetic tree of the 399 animal training and test set RF1, RF2, and RF3 model isolates. Innermost annotation ring (Serovar) specifies whether an isolate was *S.* Typhimurium or a monophasic variant of *S.* Typhimurium, second annotation ring (DT104 outbreak link) specifies whether an isolate exhibited the DT104 outbreak specific SNP address of 60.11.15.16.458.459.x, third annotation ring (ML model dataset) specifies whether an isolate belonged to the training or the test machine learning model dataset, fourth annotation ring (Host) specifies the primary source class of each of the isolates, and fifth annotation ring (BAPS level 1) specifies the BAPS cluster each isolate was assigned to at the first hierarchical level. Bootstrap branch support values between 80% and 100% are shown on the tree. The tree is rooted at the outgroup strain SRR8820637.

Phylogenetic analysis of the 399 animal isolates revealed varying degrees of genetic relatedness (Figure 1). As expected, the 60 DT104 outbreak related isolates (SNP address 60.11.15.16.458.459.x) clustered in a single clade of the phylogenetic tree (Figure 1). All 60 isolates were assigned to BAPS cluster 1 together with another 80 isolates at the first hierarchical level of the BAPS clustering algorithm. The 140 BAPS cluster 1 isolates were genetically identical at the 100 SNP threshold and they occupied neighbouring clades on the phylogenetic tree (Figure 1). At the second hierarchical level of BAPS, the 60 DT104 outbreak related isolates were all assigned to a single cluster not shared with other isolates. The 322 training and 77 test set isolates (Table 1) were evenly distributed throughout the phylogenetic tree (Figure 1).

There was reasonably well defined clustering by host type amongst the 399 animal isolates (Figure 1). The Pigs isolates were largely confined to two phylogenetic tree clades that overlapped with BAPS clusters 4 and 3 (Figure 1). BAPS cluster 4 comprised almost exclusively monophasic *S.* Typhimurium isolates. BAPS cluster 6 comprised Sheep isolates, BAPS cluster 5 largely comprised Game isolates, and BAPS cluster 1 isolates were mostly from the Cattle, OtherMammals, and Sheep primary sources (Figure 1).

**Figure 2:**
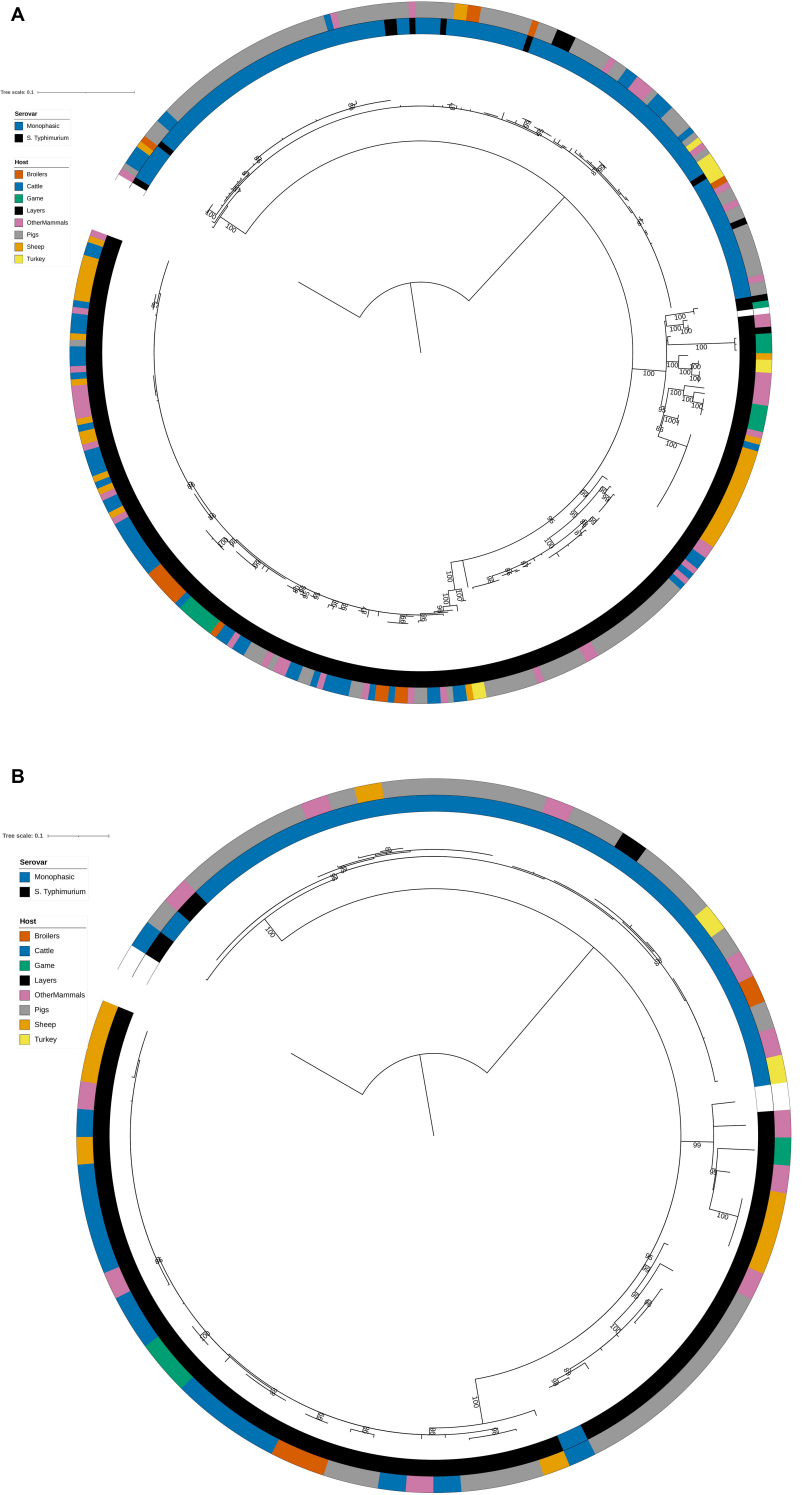
**(A)** Phylogenetic tree of the 322 animal training set RF1, RF2, and RF3 model isolates constructed from variable sites from the 163 cgMLST loci retained as machine learning model inputs. Innermost annotation ring (Serovar) specifies whether an isolate was *S.* Typhimurium or a monophasic variant of *S.* Typhimurium, and second annotation ring (Host) specifies the primary source class of each of the isolates. Bootstrap branch support values between 80% and 100% are shown on the tree. The tree is rooted at the outgroup strain SRR8820637. **(B)** Phylogenetic tree of the 77 animal test set RF1, RF2, and RF3 model isolates constructed from variable sites from the 163 cgMLST loci retained as machine learning model inputs. Innermost annotation ring (Serovar) specifies whether an isolate was *S.* Typhimurium or a monophasic variant of *S.* Typhimurium, and second annotation ring (Host) specifies the primary source class of each of the isolates. Bootstrap branch support values between 80% and 100% are shown on the tree. The tree is rooted at the outgroup strain SRR8820637.

The structures of both the animal isolate training (Figure 2A) and test (Figure 2B) set phylogenetic trees based on the variable sites from the 163 cgMLST loci used as model features were highly concordant with the tree constructed from the core genome variable sites (Figure 1). Thus, selection of model features was not biased to specific primary sources.

### 3.2 Classification of the human isolates

The hyperparameter tuned RF1, RF2, and RF3 models were highly congruent in their ability to correctly predict the primary source classes of the training and the test set animal isolates as evidenced by the highly similar training and test set accuracy and kappa values produced by these models (Table 2). Details of the assignments of the training and the test set isolates to the different sources by the tuned RF1, RF2, RF3 models that led to the selection of the tuned RF1 model (with *mtry* = 109; Tables 3, 4) for prediction of the primary sources of 662 human isolates are provided in the Supplementary material and the Supplementary Tables S2–S5.

**Table 3:** The tuned RF1 machine learning model confusion matrix for the assignment of 322 training set animal origin *S.* Typhimurium and monophasic *S.* Typhimurium isolates to eight primary source classes.

	Broilers	Cattle	Game	Layers	OtherMammals	Pigs	Sheep	Turkey
Broilers	**15**	1	0	0	0	0	0	0
Cattle	0	**60**	0	0	7	1	8	0
Game	0	0	**14**	0	0	0	0	0
Layers	0	0	0	**5**	0	0	0	0
OtherMammals	0	0	0	0	**36**	0	1	0
Pigs	1	0	0	1	2	**131**	0	0
Sheep	0	1	0	0	0	0	**29**	0
Turkey	0	0	0	0	0	0	0	**9**
Sensitivity	0.938	0.968	1.000	0.833	0.800	0.992	0.763	1.000
Specificity	0.997	0.939	1.000	1.000	0.996	0.979	0.996	1.000
Balanced accuracy	0.967	0.953	1.000	0.917	0.898	0.986	0.880	1.000

The values along the diagonal (in bold) indicate the number of isolates correctly assigned by the model to their actual primary source class. The values above and below the diagonal indicate the number of isolates incorrectly classed by the model not to their actual primary source class (column headers) but to the model predicted source (row names). The isolates were assigned to the primary source class with the highest model computed probability of assignment. Balanced accuracy is the average of the sensitivity (true positive rate) and specificity (true negative rate) values for each primary source class.

**Table 4:** The tuned RF1 machine learning model confusion matrix for the assignment of 77 test set animal origin *S.* Typhimurium and monophasic *S.* Typhimurium isolates to eight primary source classes.

	Broilers	Cattle	Game	Layers	OtherMammals	Pigs	Sheep	Turkey
Broilers	**2**	0	0	0	0	0	0	0
Cattle	0	**13**	0	0	1	2	4	0
Game	0	0	**3**	0	0	0	0	0
Layers	0	0	0	**0**	0	0	0	0
OtherMammals	0	0	0	0	**5**	0	0	0
Pigs	1	2	0	1	5	**30**	0	0
Sheep	0	0	0	0	0	0	**5**	0
Turkey	0	0	0	0	0	1	0	**2**
Sensitivity	0.667	0.867	1.000	0.000	0.455	0.909	0.556	1.000
Specificity	1.000	0.887	1.000	1.000	1.000	0.796	1.000	0.987
Balanced Accuracy	0.833	0.877	1.000	0.500	0.727	0.852	0.778	0.993

The values along the diagonal (in bold) indicate the number of isolates correctly assigned by the model to their actual primary source class. The values above and below the diagonal indicate the number of isolates incorrectly classed by the model not to their actual primary source class (column headers) but to the model predicted source (row names). The isolates were assigned to the primary source class with the highest model computed probability of assignment. Balanced accuracy is the average of the sensitivity (true positive rate) and specificity (true negative rate) values for each primary source class.

Applying the RF1 model to the 322 animal training set isolates, 4.7% isolates sampled from animal sources, 8.7% sampled from farm environment, and 14.9% sampled from sources of unspecified origin were incorrectly assigned to their actual primary source class. Of the 77 animal test set isolates, 17.0% sampled from animal sources, and 40.0% sampled from sources of unspecified origin were incorrectly assigned, whereas 100.0% of the farm environment isolates were correctly assigned. Therefore, assignment of isolates obtained from sources of unspecified origin had the lowest accuracy. For the entire animal isolate dataset, there were 18 incorrectly assigned isolates sampled from sources of unspecified origin, the majority of which (*n* = 13) were OtherMammals isolates incorrectly assigned to the Cattle or Pigs primary source classes. Five of these 13 isolates, all incorrectly assigned to the Cattle, were DT104 outbreak related (SNP address: 60.11.15.16.458.459.x) and hence it is possible that this was the reason why the RF1 model misassigned those five isolates and not because they were sampled from sources of unspecified origin. Interestingly, of the other eight of the 13 incorrectly assigned OtherMammals isolates, one was assigned to Cattle and seven assigned to Pigs. This result indicated that the companion animals could possibly be acting as secondary hosts that were infected by the farm animals. Furthermore, on 13 different occasions when the farm environment isolates and isolates sampled from sources of unspecified origin were collected from the same premises as the animal source isolates, the unknown sampling type isolates shared the same SNP address up to the 5 SNP threshold with isolates of known provenance. Thus, for example, a Pigs farm environment isolate sampled from rat faeces shared a SNP address with three isolates sampled from the same premise from Pigs (and all were assigned to the Pigs primary source class). In the entire animal isolate dataset, only two isolates that were sourced from the farm environment were assigned by the RF1 model to an incorrect primary source. Overall, the results of the RF1 model were acceptable even if the provenance of all isolates included in the animal training and test sets was not 100% clear.

**Figure 3:**
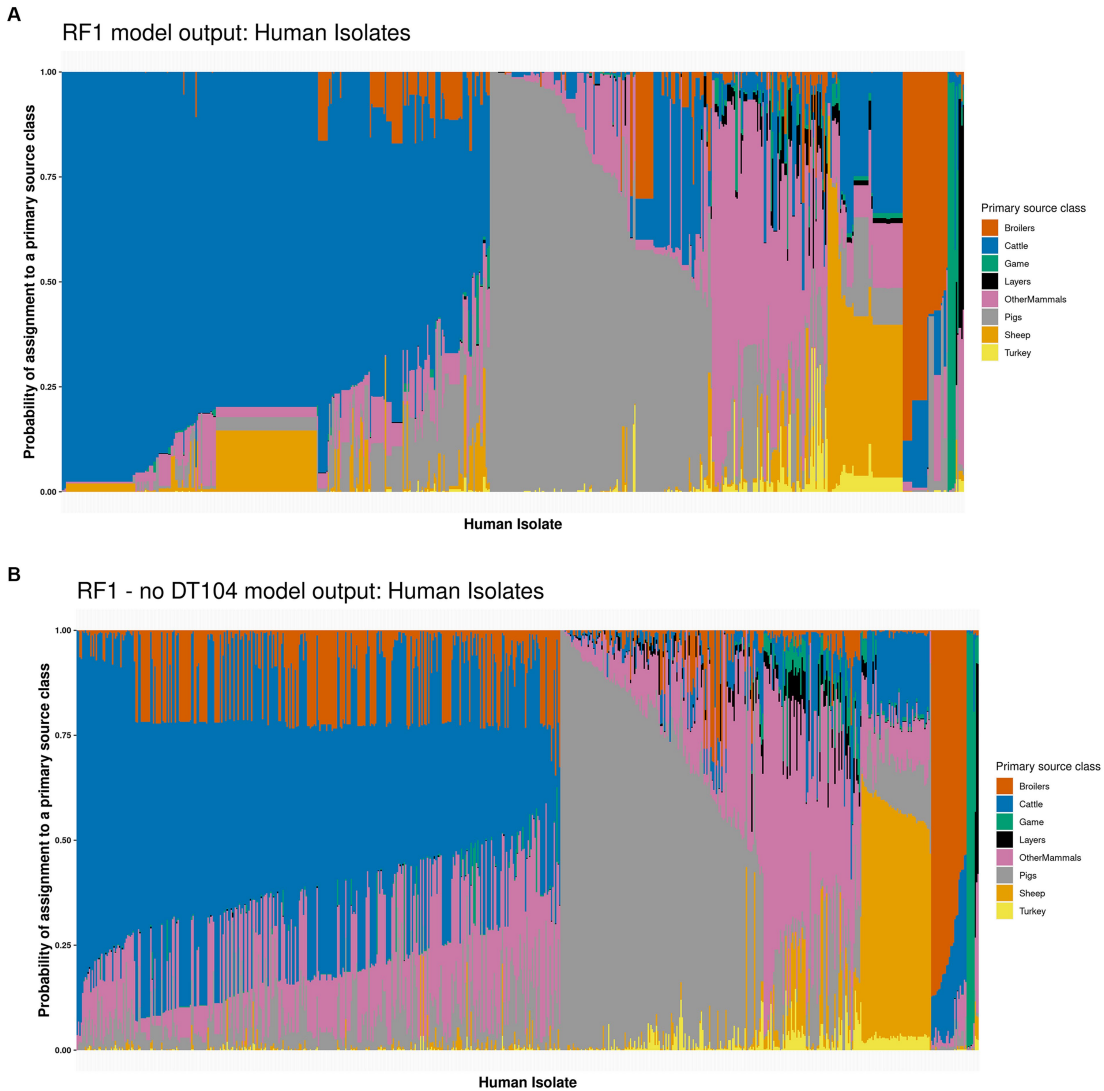
**(A)** The tuned RF1 model based assignment of 662 human isolates to eight primary source classes. The isolates are ordered by their probability of assignment to the Cattle (314 assigned isolates), followed by the Pigs (163 assigned isolates), OtherMammals (85 assigned isolates), Sheep (55 assigned isolates), Broilers (33 assigned isolates), Game (eight assigned isolates), and Layers (four assigned isolates) primary sources. **(B)** The tuned RF1—no DT104 model based assignment of 662 human isolates to eight primary source classes. The isolates are ordered by their probability of assignment to the Cattle (355 assigned isolates), followed by the Pigs (149 assigned isolates), OtherMammals (72 assigned isolates), Sheep (51 assigned isolates), Broilers (26 assigned isolates), Game (seven assigned isolates), and Layers (two assigned isolates) primary sources. No human isolates were assigned to the Turkey primary source class by either of the two models. Each vertical bar represents a single human isolate. The colour composition of each bar reflects the probability of assignment of an isolate to each of the eight primary sources. The more uniform the colour the higher the probability of assignment of an isolate to a single primary source class.

Applying the tuned RF1 model to predict the sources of 662 human isolates indicated that 314 (47.4%) isolates were attributed to Cattle, 163 (24.6%) to Pigs, 85 (12.8%) to OtherMammals, 55 (8.3%) to Sheep, 33 (5.0%) to Broilers, 8 (1.2%) to Game, and 4 (0.6%) to Layers with a highest probability of assignment value (Figure 3A and Supplementary Table S6). Sum of the probabilities of assignment for each primary source class indicated that 288 (43.7%) human isolates were assigned to Cattle, 167 (25.2%) to Pigs, 89 (13.4%) to OtherMammals, 49 (7.4%) to Sheep, 42 (6.4%) to Broilers, 12 (1.8%) to Game, 8 (1.2%) to Layers, and 7 (1.1%) to Turkey (Supplementary Table S6). The probability of assignment for 111 (16.8%) human isolates to a primary source class was low, below 0.500. Forty five of the 111 low confidence probability of assignment human isolates were assigned to Sheep and 32 to OtherMammals (Figure 3A and Supplementary Table S6).

**Figure 4:**
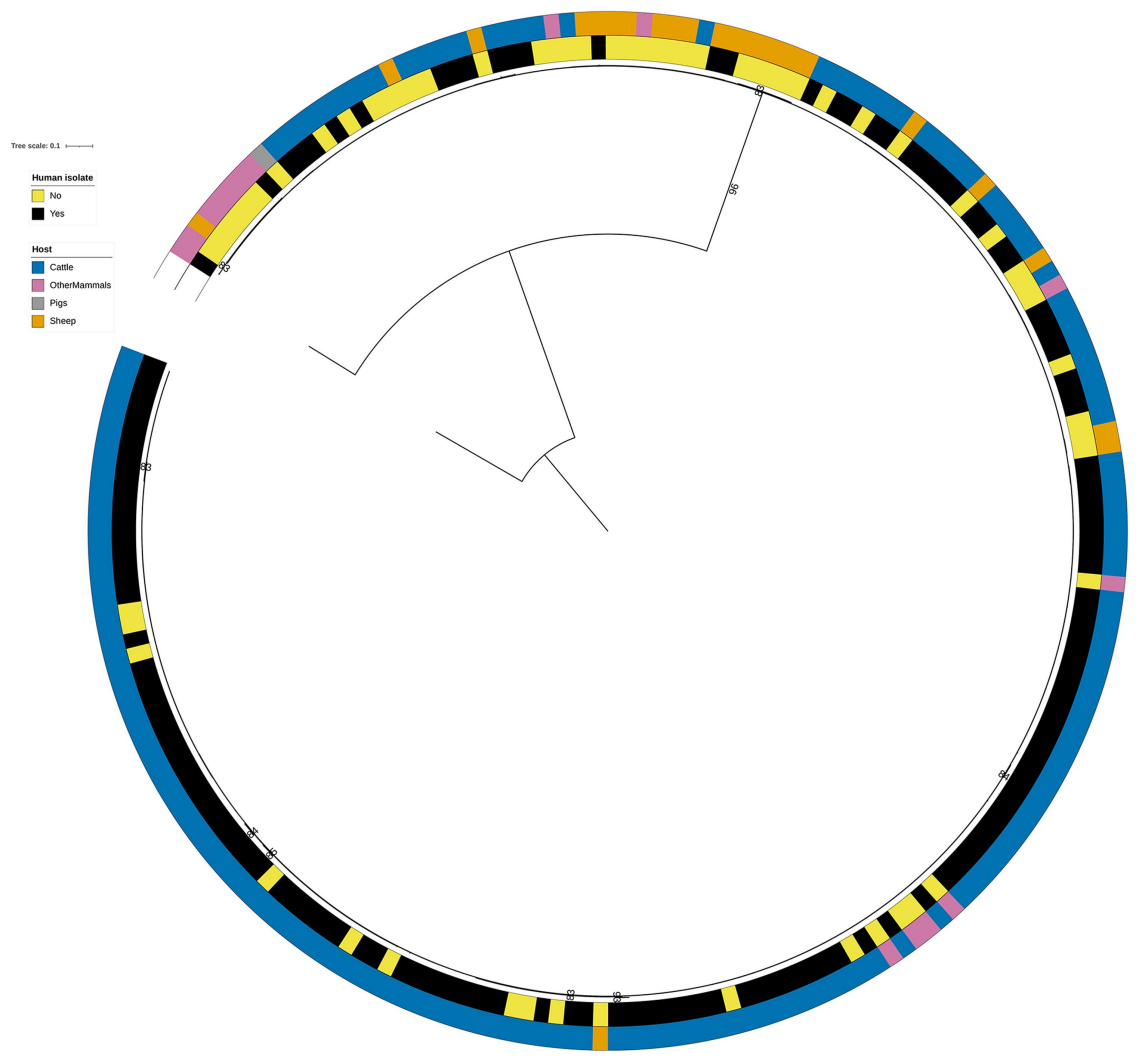
Phylogenetic tree of the 60 animal and 141 human RF1, RF2, and RF3 model *S.* Typhimurium isolates that were linked to the DT104 outbreak via the 60.11.15.16.458.459.x SNP address. Innermost annotation ring (Human isolate) specifies whether an isolate was collected from a human salmonellosis patient, and second annotation ring (Host) specifies the primary source class of each of the animal isolates and the RF1 assigned primary source of each of the human isolates. Bootstrap branch support values between 80% and 100% are shown on the tree. The tree is rooted at the outgroup strain SRR8820637.

Overall, the RF1 generated assignments of human isolates to animal primary sources correlated well with the known epidemiological data. In this dataset, 141 (21.3%) of the 662 human isolates were related to the *S.* Typhimurium DT104 outbreak that was primarily linked to the consumption of beef and mutton that became contaminated due to poor farm and abattoir practices (Figure 4). Of these, 136 (96.5%) isolates were assigned to Cattle, 3 (2.1%) to Sheep, and 2 (1.4%) to OtherMammals (Figure 4 and Supplementary Table S6). Therefore, out of a total of 314 human isolates that were assigned to Cattle, 136 (43.3%) were the DT104 outbreak related isolates. There was a marked contrast in how confidently the tuned RF1 model assigned these two groups of human isolates (the DT104 outbreak related isolates and the remainder) to the Cattle and Sheep primary sources. All 139 DT104 outbreak related human isolates were assigned to either Cattle or Sheep with high confidence probability of assignment values of above 0.500. By contrast, of the 178 human isolates with different SNP address assigned to Cattle, 15 (8.4%) were assigned with a low confidence probability of assignment of below 0.500. Equally, of the 52 human isolates not related to the DT104 outbreak assigned to Sheep, 45 (86.5%) were assigned with a low confidence probability of assignment (Figure 3A and Supplementary Table S6). Additionally, of the 314 human isolates that the tuned RF1 model assigned to Cattle, 311 (99.0%) were of DT104 (including isolates with different SNP address to the DT104 outbreak related isolates), and of the 55 isolates assigned to Sheep, 47 (85.5%) isolates were of DT104 (including isolates with different SNP address to the DT104 outbreak related isolates) (Supplementary Table S6). Taken together, the above results strongly indicate that the human isolates for which there was epidemiological primary source data were assigned with high confidence to the expected primary source classes. Therefore, these outputs gave credence to the overall performance of the RF1 model.

Furthermore, 214 human isolates that were not part of the DT104 outbreak were previously analysed as part of the Horizon2020 COMPARE project (Munck et al., 2020b) where the Pigs primary source class was found to be the largest contributor to human infection (Arnold et al., 2021). The outputs of RF1 correlated with this finding as of the 163 human isolates assigned to Pigs, 126 (77.3%) were the COMPARE project isolates (Supplementary Table S6).

### 3.3 Feature importance for RF1

The top 15 RF1 model features (cgMLST loci), ranked in accordance with mean decrease in accuracy, are presented in Table 5. cgMLST locus STMMW_21601 was the feature that ranked the highest overall. STMMW_21601 was the most important feature for correct classification of the Cattle, Pigs, Sheep, and Broilers primary sources. For OtherMammals it was the fifth most important feature, for Layers the fourth most important feature, and for Turkey and Game the second most important feature. Locus STMMW_21601 represents gene *yegO*, a multidrug transporter subunit MdtC (Table 5).

**Table 5:** The top 15 RF1 model features (cgMLST loci) ranked by the mean decrease in accuracy—an overall feature importance measure across all eight primary source classes (MeanDecreaseAccuracy).

cgMLST locus	Cattle	OtherMammals	Pigs	Sheep	Broilers	Layers	Turkey	Game	MeanDecreaseAccuracy	Gene name	Gene function description
STMMW_21601	0.194	0.010	0.178	0.157	0.147	0.065	0.116	0.237	0.151	*yegO*	Multidrug transporter subunit MdtC
STMMW_04461	0.067	−0.004	0.106	0.052	0.041	0.049	0.053	0.071	0.069	*sbmA*	Microcin B17 transporter
STMMW_09941	0.057	0.005	0.042	0.033	0.035	0.026	0.033	0.039	0.037	*rec2*	ComEC family protein
STMMW_31261	0.023	−0.016	0.058	0.028	0.019	0.012	0.031	0.037	0.032		Sodium: sulfate symporter
STMMW_17951	0.009	−0.012	0.066	0.004	0.001	0.020	0.032	0.002	0.029	*dadA*	D-amino acid dehydrogenase small subunit
STMMW_02471	0.078	0.009	0.015	−0.003	0.032	0.008	0.014	0.020	0.025	*cutF*	Lipoporotein NlpE
STMMW_00981	0.029	0.013	0.002	0.016	0.023	0.001	0.010	0.314	0.024	*imp*	LPS assembly protein LptD
STMMW_22931	0.042	0.014	0.003	0.022	0.037	−0.004	0.013	0.169	0.023	*yojN*	Putative regulator YojN
STMMW_23721	0.102	−0.040	0.007	0.015	0.048	0.001	0.016	0.018	0.022	*yfcH*	Epimerase
STMMW_24491	0.109	−0.047	0.014	0.006	0.008	0.001	0.010	0.004	0.022	*cysK*	Cysteine synthase A
STMMW_17181	0.018	−0.002	0.031	0.021	0.021	0.015	0.022	0.042	0.022	*trpE*	Anthranilate synthase component 1
STMMW_30971	0.007	0.002	0.047	0.001	0.002	0.012	0.004	0.000	0.022	*uxaC*	Uronate isomerase
STMMW_32791	0.007	0.002	0.039	0.001	0.001	0.019	0.081	0.004	0.020	*mtr*	Probable amino acid permease
STMMW_13951	0.016	0.001	0.028	0.019	0.019	0.000	0.020	0.029	0.020	*orf242*	Helix-turn-helix-type transcriptional regulator
STMMW_18131	0.001	0.000	0.046	0.000	0.002	0.010	0.014	0.000	0.019	*yoaA*	ATP-dependent helicase

The feature importance for each of the eight primary sources the RF1 model was trained to recognize (Cattle, OtherMammals, Pigs, Sheep, Broilers, Layers, Turkey, Game) is also specified. The EnteroBase derived gene name and gene function description is specified for each of the 15 features. Note that the top ranking feature for a specific primary source may not be shown in the table as the top 15 cgMLST loci are ordered by MeanDecreaseAccuracy.

### 3.4 RF1—no DT104 model performance

The tuned RF1—no DT104 model, which was run without 60 DT104 outbreak related animal isolates, produced training set accuracy of 0.989 (95% CI: 0.969–0.998) and kappa of 0.985 (Table 2). RF1—no DT104 incorrectly assigned 3/275 training set isolates (Table 6). The test set accuracy of the tuned RF1—no DT104 model was 0.781 (95% CI: 0.660–0.875) and kappa 0.663, values that were comparable to the test set accuracy and kappa produced by the tuned RF1 model (Table 2). In total, 14 of the RF1—no DT104 test set isolates were assigned by that model to an incorrect primary source class (Table 7). Four of these isolates were also part of the test set for the RF1 model and all four were assigned to incorrect primary sources by that model.

**Table 6:** The tuned RF1—no DT104 machine learning model confusion matrix for the assignment of 275 training set animal origin *S.* Typhimurium and monophasic *S.* Typhimurium isolates to eight primary source classes, excluding all isolates with the clonal DT104 outbreak SNP address of 60.11.15.16.458.459.x.

	Broilers	Cattle	Game	Layers	OtherMammals	Pigs	Sheep	Turkey
Broilers	**15**	1	0	0	0	0	0	0
Cattle	0	**40**	0	0	0	0	0	0
Game	0	0	**14**	0	0	0	0	0
Layers	0	0	0	**6**	0	0	0	0
OtherMammals	0	0	0	0	**35**	0	0	0
Pigs	1	0	0	0	0	**132**	0	1
Sheep	0	0	0	0	0	0	**22**	0
Turkey	0	0	0	0	0	0	0	**8**
Sensitivity	0.938	0.976	1.000	1.000	1.000	1.000	1.000	0.889
Specificity	0.996	1.000	1.000	1.000	1.000	0.986	1.000	1.000
Balanced accuracy	0.967	0.988	1.000	1.000	1.000	0.993	1.000	0.944

The values along the diagonal (in bold) indicate the number of isolates correctly assigned by the model to their actual primary source class. The values above and below the diagonal indicate the number of isolates incorrectly classed by the model not to their actual primary source class (column headers) but to the model predicted source (row names). The isolates were assigned to the primary source class with the highest model computed probability of assignment. Balanced accuracy is the average of the sensitivity (true positive rate) and specificity (true negative rate) values for each primary source class.

**Table 7:** The tuned RF1—no DT104 machine learning model confusion matrix for the assignment of the 64 test set animal origin *S.* Typhimurium and monophasic *S.* Typhimurium isolates to eight primary source classes, excluding all isolates with the clonal DT104 outbreak SNP address of 60.11.15.16.458.459.x.

	Broilers	Cattle	Game	Layers	OtherMammals	Pigs	Sheep	Turkey
Broilers	**2**	0	0	0	0	0	0	0
Cattle	0	**6**	0	0	0	0	0	0
Game	0	0	**3**	0	0	0	0	0
Layers	0	0	0	**1**	0	0	0	0
OtherMammals	0	1	0	0	**4**	1	1	0
Pigs	1	3	0	0	4	**30**	1	1
Sheep	0	0	0	0	0	1	**3**	0
Turkey	0	0	0	0	0	0	0	**1**
Sensitivity	0.667	0.600	1.000	1.000	0.500	0.938	0.600	0.500
Specificity	1.000	1.000	1.000	1.000	0.946	0.688	0.983	1.000
Balanced accuracy	0.833	0.800	1.000	1.000	0.723	0.813	0.792	0.750

The values along the diagonal (in bold) indicate the number of isolates correctly assigned by the model to their actual primary source class. The values above and below the diagonal indicate the number of isolates incorrectly classed by the model not to their actual primary source class (column headers) but to the model predicted source (row names). The isolates were assigned to the primary source class with the highest model computed probability of assignment. Balanced accuracy is the average of the sensitivity (true positive rate) and specificity (true negative rate) values for each primary source class.

Of the 275 animal training set isolates, 1.0% sampled from animal sources and 1.9% sampled from sources of unspecified origin were incorrectly assigned by the RF1—no DT104 model. For the 64 test set animal isolates, 18.2% obtained from animal sources and 41.2% of isolates obtained from sources of unspecified origin were attributed to an incorrect primary source class by the RF1—no DT104 model. All training and test set isolates sampled from the farm environment were assigned correctly.

There were broad similarities in how the tuned RF1 (Figure 3A) and RF1—no DT104 (Figure 3B) models classed the 662 human isolates (Supplementary Table S6). RF1—no DT104 assigned 355 (53.6%) human isolates to Cattle, 149 (22.5%) to Pigs, 72 (10.9%) to OtherMammals, 51 (7.7%) to Sheep, 26 (3.9%) to Broilers, 7 (1.1%) to Game, and 2 (0.3%) to Layers (Figure 3B and Supplementary Table S6). Attributing human isolates to primary source classes based on the sum of probabilities of assignment for each class revealed that 240 (36.2%) human isolates were assigned to Cattle, 156 (23.6%) to Pigs, 129 (19.5%) to OtherMammals, 68 (10.3%) to Broilers, 40 (6.0%) to Sheep, 14 (2.2%) to Game, 8 (1.2%) to Layers, and 7 (1.0%) to Turkey (Supplementary Table S6). All but one of the 141 DT104 outbreak related (SNP address: 60.11.15.16.458.459.x) human isolates were assigned by the RF1—no DT104 model to the Cattle primary source class; the one isolate was assigned to OtherMammals (according to the RF1 model output, that isolate was assigned to Cattle) (Supplementary Table S6). RF1—no DT104 model assigned 125/662 (18.9%) human isolates to a primary source with a low confidence probability of assignment of below 0.500. This included 64/125 (51.2%) isolates assigned to Cattle, 40/125 (32.0%) to OtherMammals, and 15/125 (12.0%) to Pigs (Figure 3B and Supplementary Table S6). Twenty one of the 125 RF1—no DT104 model low confidence probability of assignment human isolates were related to the DT104 outbreak (Supplementary Table S6). Of the 355 human isolates that the tuned RF1—no DT104 model assigned to Cattle, 354 (99.7%) were of DT104 (including isolates with different SNP address to the DT104 outbreak related isolates), and of the 51 isolates assigned to Sheep, 44 (86.3%) isolates were of DT104 (including isolates with different SNP address to the DT104 outbreak related isolates) (Supplementary Table S6). Of the 149 human isolates assigned to the Pigs primary source by the RF1—no DT104 model, 146 (97.9%) were the COMPARE project isolates that were analysed as part of a previous study which found Pigs to be the main contributor to human infection (Arnold et al., 2021) (Supplementary Table S6). Therefore, these classification patterns were highly congruent with how these isolates were classed by the RF1 model and overlapped closely with the expectations based on the epidemiological data or outputs of other studies.

cgMLST locus STMMW_21601 was also the overall most important feature for the RF1—no DT104 model (Table 8).

**Table 8:** The top 15 RF1—no DT104 model features (cgMLST loci) ranked by the mean decrease in accuracy—an overall feature importance measure across all eight primary source classes (MeanDecreaseAccuracy).

cgMLST locus	Cattle	OtherMammals	Pigs	Sheep	Broilers	Layers	Turkey	Game	MeanDecreaseAccuracy	Gene name	Gene function description
STMMW_21601	0.063	0.078	0.096	0.006	0.003	0.048	0.075	0.045	0.051	*yegO*	Multidrug transporter subunit MdtC
STMMW_41061	0.044	0.046	0.080	0.014	−0.001	0.053	0.059	0.039	0.045	*rpoB*	DNA-directed RNA polymerase subunit beta
STMMW_04461	0.039	0.052	0.057	0.008	0.005	0.034	0.058	0.034	0.035	*sbmA*	Microcin B17 transporter
STMMW_17181	0.034	0.031	0.044	0.014	−0.004	0.034	0.041	0.034	0.029	*trpE*	Anthranilate synthase component 1
STMMW_31261	0.017	0.023	0.037	0.006	−0.008	0.037	0.033	0.020	0.026		Sodium: sulfate symporter
STMMW_17881	0.020	0.032	0.019	0.026	0.009	0.024	0.025	0.029	0.023	*treA*	“Alpha, alpha-trehalase”
STMMW_13951	0.025	0.019	0.040	0.005	0.001	0.025	0.034	0.015	0.022	*orf242*	Helix-turn-helix-type transcriptional regulator
STMMW_23721	0.054	0.094	0.029	0.001	−0.019	0.009	0.005	0.013	0.021	*yfcH*	Epimerase
STMMW_09941	0.026	0.037	0.033	0.005	0.002	0.017	0.029	0.012	0.020	*rec2*	ComEC family protein
STMMW_17951	0.002	0.005	0.004	0.007	−0.010	0.038	0.005	0.017	0.019	*dadA*	D-amino acid dehydrogenase small subunit
STMMW_00981	0.018	0.021	0.173	0.000	0.007	0.005	0.016	0.021	0.018	*imp*	LPS assembly protein LptD
STMMW_22931	0.022	0.029	0.104	−0.003	0.007	0.004	0.018	0.012	0.015	*yojN*	Putative regulator YojN
STMMW_04281	0.016	0.017	0.026	0.003	0.000	0.015	0.020	0.008	0.014	*res*	Type III restriction-modification system StyLTI enzyme res
STMMW_30971	0.005	0.012	0.002	0.008	0.003	0.022	0.002	0.005	0.014	*uxaC*	Uronate isomerase
STMMW_23551	0.043	0.052	0.026	0.000	−0.007	0.005	0.004	0.007	0.013	*yfbS*	Transcriptional regulator

The feature importance for each of the eight primary sources the RF1—no DT104 model was trained to recognize (Cattle, OtherMammals, Pigs, Sheep, Broilers, Layers, Turkey, Game) is also specified. The EnteroBase derived gene name and gene function description is specified for each of the 15 features. Note that the top ranking feature for a specific primary source may not be shown in the table as the top 15 cgMLST loci are ordered by MeanDecreaseAccuracy.

## 4 Discussion

In this study, utilising 163 cgMLST loci as model features, three distinct RF models were trained, tuned, and evaluated on 399 *S.* Typhimurium and monophasic *S.* Typhimurium animal isolates. Subsequently, the best performing model was applied to predict primary source of 662 *S.* Typhimurium and monophasic *S.* Typhimurium human clinical cases. Supervised classification algorithms, including RF, exhibit properties highly suited for attribution of foodborne pathogens. Such models first learn to associate patterns in the provided genomic data of, for instance, *S.* Typhimurium isolates originating from different primary sources (i.e., animal host species) with a specific source. Subsequently, when applied to predict the sources of human clinical cases, the algorithm will seek out the genomic data patterns it had previously learnt to recognize and use that information to assign a primary source to each of the analysed human isolates (Munck et al., 2020a). The more data a ML algorithm has been exposed to, the more accurate it should become in making such predictions, as it has the ability to learn from the patterns in the data and hence to improve its decision making capabilities (Libbrecht and Noble, 2015).

The majority of the training set isolates (*n* = 322) used in the three RF models were from the following primary sources: Pigs (41.0%), Cattle (19.3%), OtherMammals (14.0%), and Sheep (11.8%). Highly unbalanced training data has previously been noted to result in a potential bias in favor of the majority class in the ML model generated predictions (Velez et al., 2007; Njage et al., 2019b). The best performing model, RF1, assigned 47.4% of the 662 *S.* Typhimurium and monophasic *S.* Typhimurium human isolates to Cattle, 24.6% to Pigs, 12.8% to OtherMammals, and 8.3% to Sheep. Thus, it cannot be concluded that the RF1 model assigned majority of the human isolates to sources which were overrepresented in the training set. While best practice is to use a balanced training dataset when implementing a RF model, this is an idealized scenario, and such datasets can be difficult to obtain. Only a proportion of the infected animals are detected by surveillance, and many primary hosts infected with *S.* Typhimurium or monophasic *S.* Typhimurium are asymptomatic and act as a reservoir of infection (Arnold et al., 2021). Furthermore, if the collection of isolates was biased towards the livestock and farm animals and there is lack of isolate collection from other potential *S.* Typhimurium and monophasic *S.* Typhimurium reservoirs, such as wild birds or animals (Skov et al., 2008), this will have a strong impact on the ability of attribution models to inform if the isolates from the rarer sources infected the human population.

In the analysed animal isolate dataset, 163 of the 399 isolates were *S.* Typhimurium (and in two cases monophasic *S.* Typhimurium) of DT104, of which 60 were clonal isolates related to the known 2015–2018 DT104 outbreak in England and Wales [Animal and Plant Health Agency (APHA), 2017]. Even though *S.* Typhimurium of DT104 can reside in numerous host species, it is considered primarily a cattle pathogen (Poppe et al., 1998). Indeed, of the 165 isolates of DT104, 145 (87.9%) were from four mammalian primary source classes: Cattle (*n* = 59), OtherMammals (*n* = 34), Pigs (*n* = 28), and Sheep (*n* = 20). The intentional inclusion of the clonal, DT104 outbreak related isolates in the RF1 model facilitated the validation of model performance by using confirmed primary sources of human infections as model inputs. Although presence of the clonal isolates may have influenced the RF1 model outputs, the proportion of the DT104 outbreak related isolates did not exceed 50% for any of the eight primary sources. Previous studies emphasised minimizing the proportion of clonal genomes in the model training set in order to avoid artificially inflating source prediction accuracy (Zhang et al., 2019). We tested the potential model confusion by running the RF1 model without the clonal DT104 isolates (the RF1—no DT104 model) and compared the model outputs. The RF1—no DT104 model performed slightly better than RF1 at the training stage (accuracy: 0.989 vs. 0.929, kappa: 0.985 vs. 0.905) and at the test stage (accuracy: 0.781 vs. 0.779, but not kappa: 0.663 vs. 0.700), however, these metrics were highly similar thus indicating the robustness of the RF1 model to the presence of clonal isolates in the training set. The value of the kappa statistic in the range of “0.61–0.80” is indicative of “substantial” model performance (Landis and Koch, 1977) and in the range of “0.40–0.75” of “fair to good” model performance (Fleiss et al., 2003). Therefore, both models performed adequately and comparably to ML attribution models described in several recently published studies (Zhang et al., 2019; Munck et al., 2020a; Tanui et al., 2022).

A closer inspection of how the training and test isolates were attributed by the RF1 and RF1—no DT104 models revealed that of the 23 RF1 model primary source misassignments at the training stage, 15 were the DT104 outbreak related isolates. The number of training set misassignments was lower for the RF1—no DT104 model with only three incorrectly assigned isolates. There were 12 DT104 outbreak related isolates in the RF1 model test set, however, of the 17 primary source class misassignments only four were the DT104 outbreak related isolates (all incorrectly assigned to Cattle). Unlike for the training set, the majority (nine) misassignments were from another primary source class (Cattle, OtherMammals, Broilers, or Layers) to Pigs. The overrepresentation of the Pigs primary source class isolates in the training set might have been the reason for these misassignments. However, all nine isolates, of which seven were monophasic *S.* Typhimurium, clustered in clades that largely comprised Pigs isolates on the 163 cgMLST locus based test set phylogenetic tree. Therefore, it was more likely that the misassignment of these isolates was due to their genetic closeness to isolates representative of the Pigs source. Similarly, of the 14 test set isolates that were misassigned by the RF1—no DT104 model, 10 (originating from Broilers, Cattle, OtherMammals, Sheep, and Turkey) were incorrectly classed as Pigs. These 10 isolates clustered with Pigs isolates on the core genome alignment based phylogenetic tree, and eight were monophasic *S.* Typhimurium. One possible explanation for the observed test set misassignments could be that the isolates from primary sources other than Pigs were classed as Pigs by both models as these isolates did in fact originate from the Pigs primary source class that cross-infected a different primary host.

The fact that both, the RF1 and RF—no DT104 models, assigned 99.0% of the 141 DT104 outbreak related human isolates to the presumed primary sources based on the known epidemiological data supported the applicability of RF to attribution of human *Salmonella* infections. Especially in the case of foodborne outbreaks, which are inherently clonal, a RF model could be applied to rapidly detect or narrow down the potential outbreak sources provided that these were represented in the model training set (Vilne et al., 2019). It is understood that the England and Wales 2015–2018 *S.* Typhimurium DT104 outbreak most likely originated from cattle, but the spread was probably due to the movement of sheep, and most human cases that were not linked to farms were likely due to mutton consumption [Animal and Plant Health Agency (APHA), 2017]. Thus, the RF1 model, for which 20 of the 48 RF1 training set DT104 outbreak related isolates were from Cattle, behaved as expected when assigning most of the DT104 outbreak related human isolates with a high confidence probability of assignment of over 0.500 to Cattle. However, although contaminated mutton was also suspected to be a source of the DT104 outbreak in humans and 16 RF1 training set DT104 outbreak related isolates were from the Sheep primary source, only three human isolates with the DT104 outbreak SNP address were assigned to Sheep by the model. Thus, it is conceivable that at least some of the human DT104 outbreak related isolates assigned by RF1 to Cattle should have instead been assigned to Sheep. Additionally, close to 25% of the RF1 training set DT104 outbreak related isolates were from the OtherMammals primary source but the RF1 model assigned only two DT104 outbreak related human isolates to OtherMammals. As the OtherMammals primary source class (dogs and horses) was not known to be epidemiologically linked to the DT104 outbreak, it was encouraging that the model assigned very few DT104 outbreak related human isolates to this source. Furthermore, a Bayesian attribution model applied to the COMPARE project isolates concluded that “pigs were found to be the main contributor to human infection for *S.* Typhimurium/monophasic *S.* Typhimurium”, with the estimate of attribution of human isolates to pigs ranging from 48.2% to 59.3% depending on which subtyping method was used (Arnold et al., 2021). RF results were highly congruent with the outputs of the Bayesian method as RF1 assigned 58.9% of the 214 COMPARE human isolates to the Pigs primary source and of all 163 human isolates assigned by RF1 to Pigs, 77.3% were the COMPARE project isolates. Isolates from the four avian primary source classes (Broilers, Layers, Game, Turkey) comprised 14.0% (45/322) of all training set isolates but only 6.8% of the human isolates were assigned to any of these four primary sources. This result is in agreement with the conclusions of Lupolova et al. (2017) that the avian *S.* Typhimurium and monophasic *S.* Typhimurium isolates are a lower public health threat in the United Kingdom.

Largely congruent attribution of the 662 human isolates by the RF1 and the RF1—no DT104 models indicated that the assignment of the DT104 outbreak related human isolates to the presumed correct primary sources according to the available epidemiological data was not dependent on the inclusion of the DT104 outbreak related isolates in the model training set. However, the RF1—no DT104 model retained 421 cgMLST loci (2.5 times more than RF1) after feature selection and hence required a longer running time compared to RF1. Additionally, the RF1—no DT104 model produced low confidence probability of assignment values for a greater proportion of human isolates (125/662) in comparison with the RF1 model (111/662), most of which were to Cattle. For the RF1 model, the largest proportion of the low confidence probability of assignment human isolates were attributed to Sheep. Further experimentation with different proportions of clonal animal isolates retained as part of the model training set will likely be useful to better understand the influence of very closely genetically related isolates on the accuracy of classification of human isolates by ML models.

There are several potential explanations for the low confidence assignments of human isolates to a primary source, one being the lack of representation in the model training set of primary sources that human isolates had originated from. For example, for RF1, 8.1% of the low confidence probability of assignment human clinical cases had potential links to travel outside of the UK. Hence, it is plausible that the contracted *S.* Typhimurium and monophasic *S.* Typhimurium strains were genetically distinct from the bacterial populations circulating in the English and Welsh primary sources that the model was trained to recognize. However, 81.3% of the travel linked human isolates were assigned by RF1 to a source with a high confidence probability of assignment of above 0.500, and furthermore, 16.6% of isolates obtained from salmonellosis patients without apparent travel history were assigned to a source with a low confidence probability. Thus, patient travel history was likely not the sole reason behind the low confidence assignments for some human isolates. Infection via imported foods (Stein and Chirilã, 2017) or human to human salmonellosis transfer (Lupolova et al., 2017, 2019) may have been an additional reason why certain human isolates carried genetic signatures that were not represented in the model training set. Additionally, 32/111 low confidence RF1 human isolate assignments were to OtherMammals. In the RF1 training set, this primary source class comprised isolates from several distinct host species that included dogs, horses, and cats, which may have been a potential source of confusion for the model. Refining the model training set by removing this primary source class deserves further investigation.

The RF1 and RF1—no DT104 models used core genome MLST loci as model features which produced robust outputs. Other studies have found that the accessory genomes of the analysed bacterial isolates were a useful source of ML model features for attribution of *S.* Typhimurium isolates (Lupolova et al., 2017; Zhang et al., 2019). If the patterns in allelic variation of MLST loci derived from accessory genomic elements were host specific, then using the accessory genome MLST loci together with, or instead of, the cgMLST loci could be a useful approach for increasing the accuracy of RF predictions. For both the RF1 and RF1—no DT104 models, cgMLST locus STMMW_21601, which encodes multidrug transporter subunit MdtC, was the most important feature for distinguishing between isolates from different sources. Transporter proteins have been shown to play an important role for host specificity in *S.* Typhimurium (Morgan et al., 2004) thus underscoring the high relevance of STMMW_21601 for source attribution RF models. There were 153 cgMLST loci used as model features by both the RF1 and RF1—no DT104 models, including 11 of the 15 highest ranked model features. Defining a robust panel of common cgMLST loci will be a vital step in applying only those features that are the most useful for differentiation of a broad selection of *S.* Typhimurium and monophasic *S.* Typhimurium primary sources.

In conclusion, the model outputs presented herein provide good support for the applicability of RF as a valid approach for attribution of bacterial zoonotic pathogens, in particular if complemented by precise epidemiological data for both the primary source and human isolates. Further optimization of the method should include expanding the training set panel of isolates to cover the less frequently encountered *S.* Typhimurium and monophasic *S.* Typhimurium reservoir hosts (i.e., hosts other than common farm and domestic animals) as well as introducing model features representative of the accessory genome of the analysed isolates. With the ever-accelerating sequencing of high quality genomic data of bacterial pathogens, both those objectives ought to be very much achievable.

## Data availability statement

The WGS data of isolates analysed in this study have been deposited on EnteroBase with the exception of sequences of 19 isolates (APHA01-APHA19 in Supplementary Table S1) that will be made available upon request on case by case basis due to the high level of sensitivity of these data.

## Ethics statement

The animal study was approved, this study uses strains obtained from routine surveillance of livestock farms, outbreak investigations, control programs, research projects, or from already existing data sets. The APHA Ethics Committee did not require the study to be reviewed or approved by an ethics committee because no animal experimentation of any kind was performed to obtain the isolates. The study was conducted in accordance with the local legislation and institutional requirements.

## Author contributions

JG: Conceptualization, Data curation, Formal analysis, Investigation, Methodology, Project administration, Software, Writing – original draft, Writing – review & editing. YT: Methodology, Software, Writing – review & editing. MC: Data curation, Supervision, Writing – original draft, Writing – review & editing. TD: Conceptualization, Data curation, Supervision, Writing – original draft, Writing – review & editing. LP: Conceptualization, Data curation, Funding acquisition, Methodology, Resources, Supervision, Writing – original draft, Writing – review & editing.

## Footnotes

^1^ https://github.com/ncbi/sra-tools/wiki/HowTo:-fasterq-dump

^2^ https://github.com/tseemann/shovill

^3^ https://github.com/tseemann/snippy
