# Supplementary material for: Development and validation of a random forest algorithm for source attribution of animal and human Salmonella Typhimurium and monophasic variants of S. Typhimurium isolates in England and Wales utilising whole genome sequencing data
Source: Front Microbiol. 2024 Mar 12;14:1254860. doi: 10.3389/fmicb.2023.1254860 (PMC10963456; doi:10.3389/fmicb.2023.1254860)
Supplement: Supplementary file 1 [file Data_Sheet_1.zip › Data Sheet.docx]

# Supplementary Data

## Materials and Methods: Recursive feature elimination (*rfe*)

Using the *rfe* classification algorithm, backwards feature selection of the 902 cgMLST loci was performed by several ranking and elimination steps to rank the features in accordance with feature importance. The least important features were sequentially deleted, the rankings recalculated, and this proceeded until all features had been eliminated. The backwards feature elimination with *rfe* was implemented via a random forest classifier using a 25-fold cross validation repeated 10 times. After completion of the *rfe* algorithm, the *pickSizeTolerance* function of caret was used to further reduce the number of the *rfe* selected features such that the model accuracy was not more than 1% worse than if all of the highest ranking features were retained. After this step, a total of 130 top-ranking features were selected for the model.

## Materials and Methods: Feature selection with *Boruta*

Using the *Boruta* feature selection algorithm, a top-down search for relevant features was performed by comparing original attributes’ importance with importance achievable at random, estimated using their permuted copies, and progressively eliminating irrelevant features to stabilise the test. The *Boruta* algorithm was run with the maxRuns parameter set to 50,000. Of the 902 features, 109 were confirmed as important. Thirty three of the features selected by the *Boruta* approach were not in the 130 *rfe* selected feature set.

## Materials and Methods: Tuning and testing random forest models for source attribution

In our approach each of the three random forest (RF) models (RF1, RF2, RF3) was first run on the 322 animal isolate training set (Table 1) to learn to associate specific patterns in the cgMLST allele calls at the 163 retained cgMLST loci (model features with the highest importance) with each of the eight primary source classes. Importantly, the data on the primary source class of each of the training set animal isolates was provided to the models to facilitate model learning. At this stage, model tuning and selection of the optimal model parameters was performed such that each of the three RF models was run multiple times – each time with a unique combination of the model tuning parameters (hyperparameters). For the RF1 and the RF2 models, both of which were run with the randomForest R package random forest algorithm, model tuning was performed by computing a model for each value of the *mtry* hyperparameter (the number of randomly selected model features at each split in the decision tree) that ranged from 1 to 163 (Table 2). For the RF3 model, which was run with the ranger R package random forest algorithm, 1,134 combinations of the *mtry* (ranging from 2 to 162 in increments of 2), *splitrule* (gini or extratrees), and *min.node.size* (1, then ranging from 5 to 30 in increments of 5) hyperparameters were tested (Table 2). For each set of hyperparameters, model accuracy was estimated by resampling the training animal data set, either using out of bag sampling (10,000 iterations – RF1) or a 10-fold cross validation repeated 10 times (RF2, RF3), and the optimal hyperparameter set was the one with the highest estimated accuracy (Table 2). Subsequently, the tuned RF1, RF2, and RF3 models were applied to predict the primary source classes of the 322 training set isolates (here, by default, high accuracies were expected since these were the data that these models were trained on) and then to predict the primary source classes of the 77 test set animal isolates (Table 1). As the primary source class of each of the test set isolates was withheld from the models, this was the true evaluation of model performance which allowed to quantify how well the models had learnt to differentiate between the different primary source classes. The models assigned each of the eight primary source classes to each of the test set isolates with a probability of assignment ranging from zero to one. The primary source class with the highest probability of assignment value was the predicted class for each isolate. The closer the highest probability of assignment value was to one the more confidence there was in the model generated primary source class predictions, whereas probability of assignment values below 0.500 were regarded as low confidence predictions. If at this stage the models assigned a satisfactory proportion of the test set isolates to their true primary source class, model performance was deemed adequate without the need for further model training and tuning.

## Results: Model training and test set outputs for the three RF models

For RF1, model tuning with 163 *mtry* hyperparameter values (1 to 163) produced accuracy values ranging from 0.602 (*mtry* = 1) to 0.786 (*mtry* = 109) (Table 2). Applying the optimal (i.e. tuned) RF1 model with *mtry* = 109 to predict the primary source classes of the training set animal isolates (Table 1) not surprisingly produced excellent training set accuracy of 0.929 (95% CI: 0.895-0.954) and a kappa of 0.905 (Table 2). Of the 23 misassignments (an isolate was assigned by the model to an incorrect primary source class, i.e. the probability of assignment of an isolate to each of the eight primary source classes was higher (and highest) to an incorrect primary source class than to the actual primary source class), 20 involved isolates from the four mammalian primary source classes (Table 3). For example, of the 45 OtherMammals isolates, 36 were assigned to the OtherMammals primary source class, seven were assigned to the Cattle primary source class, and two were assigned to the Pigs primary source class (Table 3). Balanced accuracy was lowest for the Sheep primary source class at 0.880 (Table 3). For the optimal RF1 model predictions performed on the test set animal isolates (Table 1), both the accuracy and kappa were lower than for the training set, at 0.779 (95% CI: 0.670-0.866) and 0.700, respectively (Table 2). This reflected the fact that of the 77 test set isolates, 17 were assigned to an incorrect primary source class. As was the case for the training set, the majority (14) of these misassignments were amongst the test set isolates originating from the different mammalian primary source classes (Table 4). The lowest balanced accuracy of 0.500 was for the Layers primary source class (the single Layers test set isolate was assigned by the model to the Pigs primary source class, but no isolates were incorrectly assigned to the Layers class) (Table 4). The second lowest balanced accuracy (0.727) was for OtherMammals, as five of these isolates were incorrectly assigned to the Pigs primary source class and one isolate was incorrectly assigned to the Cattle primary source class (Table 4).

Hyperparameter tuning of the RF2 model produced accuracy values ranging from 0.603 (*mtry* = 1) to 0.775 (*mtry* = 49) for 163 different *mtry* hyperparameter values (Table 2). The optimal RF2 model, with *mtry* = 49, exhibited high training set accuracy (0.901, 95% CI: 0.863-0.931) and kappa (0.867) (Table 2) when applied to predict the primary source classes of the training set animal isolates. In total, 32 training set isolates were assigned by the RF2 model to an incorrect primary source class. Of these, 28 misassignments were amongst isolates from the different mammalian primary source classes: Cattle, OtherMammals, Pigs, Sheep (Supplementary Table 2A). For the training set RF2 model predictions, balanced accuracy was lowest for the OtherMammals primary source class (0.843), as eight of these isolates were assigned by the model to the Cattle primary source class, five isolates were assigned to the Pigs primary source class, one isolate was assigned to the Sheep primary source class, and one Sheep isolate was incorrectly assigned to the OtherMammals primary source class (Supplementary Table 2A). Applying the optimal RF2 model to predict the primary source classes of the test set animal isolates resulted in the test set accuracy of 0.805 (95% CI: 0.699-0.887), whereas kappa was 0.727 (Table 2). There was a total of 15 misassignments, of which 12 were amongst the four mammalian primary source classes (Supplementary Table 2B). The Layers primary source class had the lowest balanced accuracy of 0.500. A single Layers test set isolate was wrongly classed as Pigs (and no test set isolates from the other primary source classes were assigned to Layers). The OtherMammals primary source classes exhibited the second lowest balanced accuracy (0.727) (Supplementary Table 2B). Five of these isolates were incorrectly assigned to the Pigs primary source class and one isolate was incorrectly assigned to the Cattle primary source class (Supplementary Table 2B).

For RF3, out of 1,134 different hyperparameter combinations tested, the optimal hyperparameters were *mtry* = 40, *splitrule* = gini, and *min.node.size* = 1, which produced accuracy of 0.778 (Table 2). Using the optimal RF3 model to predict the primary source classes of the training set animal isolates produced accuracy and kappa values of 0.913 (95% CI: 0.877-0.941) and 0.884, respectively (Table 2). In total there were 28 misassignments for the training set isolates, 24 of which were amongst the mammalian classes (Supplementary Table 3A). Balanced accuracy was, at 0.896, the lowest for the Sheep primary source class as eight Sheep training set isolates were classed by the model as Cattle and one was classed as OtherMammals. In addition, two Cattle isolates and one OtherMammals isolate were incorrectly assigned to the Sheep primary source class (Supplementary Table 3A). For the test set predictions (Table 2), the outputs of the optimal RF3 model (Supplementary Table 3B) were identical to the test set predictions produced by the optimal RF2 model described above (Supplementary Table 2B).

## Results: Selection of RF1 as the model to predict the primary sources of 662 human isolates

Overall, there were only minor differences in how the three random forest models: RF1, RF2, and RF3, classed the training and the test set animal isolates (Table 2). Detailed examination of the outputs of these three models, and in particular focusing on those animal isolates that were assigned by the models to an incorrect primary source class, led us to select the RF1 model as the “best” of these three models. Hence this was the model that was applied to predict the primary source classes of the 662 human isolates.

For the majority of the animal isolates that were assigned correctly to their actual primary source class by all three models, RF1 produced the highest probability of assignment of an isolate to its actual primary source class, followed by RF2 (though for some isolates it was RF2 that produced the highest probability of assignment to the correct primary source class), and then RF3 (which for certain isolates produced the highest probability of assignment to the correct primary source class). RF3, when correctly assigning an isolate to its actual primary source class, tended to “spread” the probability of assignment values between several primary source classes (in particular, between the four mammalian primary source classes). For example, if the actual primary source class of an isolate was Cattle, RF1 (and RF2) assigned that isolate with a probability of assignment of 0.950 to Cattle and 0.050 to Sheep, whereas for the same isolate RF3 produced a probability of assignment of 0.600 to Cattle, 0.300 to Sheep, and 0.100 to OtherMammals.

A similar observation was made for the isolates that were assigned to the same incorrect primary source class by all three models (Supplementary Table 4 and 5). RF1 was the most “confident” in such incorrect assignments (i.e. RF1 produced the highest probability of assignment to an incorrect primary source class in comparison with the other two models), followed by RF2, and then RF3. However, this difference in model confidence when assigning an isolate to an incorrect primary source class was not as obvious between RF1 and RF2 as for a substantial number of incorrectly assigned isolates it was RF2 that produced the highest probability of assignment to an incorrect primary source class (Supplementary Table 4 and 5). RF3 usually produced the lowest incorrect probability of assignment (and hence the highest probability of assignment to the correct primary source class – but still lower than the highest incorrect probability of assignment). Thus, as was the case when isolates were assigned to the correct primary source class by all three models, RF3 tended to spread the probability of assignment values between several primary source classes also when an isolate was assigned to an incorrect primary source class (Supplementary Table 4 and 5).

RF1 was the best of the three models when applied to predict the primary source classes of the training set isolates as it assigned the highest proportion of the training set isolates to their actual primary source class. There were nine training set isolates which were assigned by RF1 to the correct primary source class but which were incorrectly assigned by both RF2 and RF3 or only by RF2 (Supplementary Table 4). For only one of those isolates (L01659-17), RF1 produced a low confidence probability of assignment to its correct primary source class of below 0.500, whereas the other eight isolates were assigned by RF1 to their correct primary source class with a reasonably confident probability of assignment of approximately 0.550 to 0.650 (Supplementary Table 4). Three of the four training set isolates that were classed incorrectly only by RF2 were assigned by RF3 to their correct primary source class but with a low confidence probability of assignment of below (or equal to) 0.500. RF1 consistently produced the highest correct (i.e. to the actual primary source class) probability of assignment values for these four isolates (Supplementary Table 4).

RF1 incorrectly classed 17 of the test set isolates (14 of these misassignments involved confusion between different mammalian primary source classes), whereas RF2 and RF3 both incorrectly classed 15 of these isolates (12 of these misassignments involved confusion between different mammalian primary source classes) (Supplementary Table 5). For the two test set isolates that were classed incorrectly only by RF1, both RF2 and RF3 produced low confidence probability of assignment values for the assignment of these isolates to a correct primary source class. In both cases, RF1 assigned these isolates to Cattle whereas their actual primary source class was Sheep. Both isolates featured the clonal DT104 outbreak SNP address (Supplementary Table 5).

Therefore, although RF1 performed slightly worse than RF2 or RF3 when assigning the test set isolates, RF1 was the best at the classification of the training set isolates. For the two cases where RF1 incorrectly assigned the test set isolates, RF2 and RF3 were not highly confident in assigning those isolates to their actual primary source classes. Thus, overall, RF1 should be selected as the best of the three models and applied to predict the primary source classes of the human isolates.

# Supplementary Figures and Tables

**Supplementary** **Table 1** Description of the isolate ID, year of collection, model supplied and predicted primary source class, and sample type for the 399 animal training and test set isolates and of the isolate ID and year of collection for the 662 human isolates.

*Excel table*

**Supplementary Table 2A** The tuned RF2 machine learning model confusion matrix for the assignment of 322 training set animal origin *S*. Typhimurium and monophasic *S*. Typhimurium isolates to eight primary source classes. The values along the diagonal (in bold) indicate the number of isolates correctly assigned by the model to their actual primary source class. The values above and below the diagonal indicate the number of isolates incorrectly classed by the model not to their actual primary source class (column headers) but to the model predicted source (row names). The isolates were assigned to the primary source class with the highest model computed probability of assignment. Balanced accuracy is the average of the sensitivity (true positive rate) and specificity (true negative rate) values for each primary source class.

|  | Broilers | Cattle | Game | Layers | OtherMammals | Pigs | Sheep | Turkey |
| --- | --- | --- | --- | --- | --- | --- | --- | --- |
| Broilers | **14** | 1 | 0 | 0 | 0 | 0 | 0 | 0 |
| Cattle | 0 | **58** | 0 | 0 | 8 | 2 | 8 | 0 |
| Game | 0 | 0 | **14** | 0 | 0 | 0 | 0 | 0 |
| Layers | 0 | 0 | 0 | **5** | 0 | 0 | 0 | 0 |
| OtherMammals | 0 | 0 | 0 | 0 | **31** | 0 | 1 | 0 |
| Pigs | 2 | 1 | 0 | 1 | 5 | **130** | 0 | 0 |
| Sheep | 0 | 2 | 0 | 0 | 1 | 0 | **29** | 0 |
| Turkey | 0 | 0 | 0 | 0 | 0 | 0 | 0 | **9** |
|  |  |  |  |  |  |  |  |  |
| Sensitivity | 0.875 | 0.936 | 1.000 | 0.833 | 0.689 | 0.985 | 0.763 | 1.000 |
| Specificity | 0.997 | 0.931 | 1.000 | 1.000 | 0.996 | 0.953 | 0.989 | 1.000 |
| Balanced Accuracy | 0.936 | 0.933 | 1.000 | 0.917 | 0.843 | 0.969 | 0.876 | 1.000 |

**Supplementary Table 2B** The tuned RF2 machine learning model confusion matrix for the assignment of 77 test set animal origin *S*. Typhimurium and monophasic *S*. Typhimurium isolates to eight primary source classes. The values along the diagonal (in bold) indicate the number of isolates correctly assigned by the model to their actual primary source class. The values above and below the diagonal indicate the number of isolates incorrectly classed by the model not to their actual primary source class (column headers) but to the model predicted source (row names). The isolates were assigned to the primary source class with the highest model computed probability of assignment. Balanced accuracy is the average of the sensitivity (true positive rate) and specificity (true negative rate) values for each primary source class.

|  | Broilers | Cattle | Game | Layers | OtherMammals | Pigs | Sheep | Turkey |
| --- | --- | --- | --- | --- | --- | --- | --- | --- |
| Broilers | **2** | 0 | 0 | 0 | 0 | 0 | 0 | 0 |
| Cattle | 0 | **13** | 0 | 0 | 1 | 2 | 2 | 0 |
| Game | 0 | 0 | **3** | 0 | 0 | 0 | 0 | 0 |
| Layers | 0 | 0 | 0 | **0** | 0 | 0 | 0 | 0 |
| OtherMammals | 0 | 0 | 0 | 0 | **5** | 0 | 0 | 0 |
| Pigs | 1 | 2 | 0 | 1 | 5 | **30** | 0 | 0 |
| Sheep | 0 | 0 | 0 | 0 | 0 | 0 | **7** | 0 |
| Turkey | 0 | 0 | 0 | 0 | 0 | 1 | 0 | **2** |
|  |  |  |  |  |  |  |  |  |
| Sensitivity | 0.667 | 0.867 | 1.000 | 0.000 | 0.455 | 0.909 | 0.778 | 1.000 |
| Specificity | 1.000 | 0.919 | 1.000 | 1.000 | 1.000 | 0.796 | 1.000 | 0.987 |
| Balanced Accuracy | 0.833 | 0.893 | 1.000 | 0.500 | 0.727 | 0.852 | 0.889 | 0.993 |

**Supplementary Table 3A** The tuned RF3 machine learning model confusion matrix for the assignment of 322 training set animal origin *S*. Typhimurium and monophasic *S*. Typhimurium isolates to eight primary source classes. The values along the diagonal (in bold) indicate the number of isolates correctly assigned by the model to their actual primary source class. The values above and below the diagonal indicate the number of isolates incorrectly classed by the model not to their actual primary source class (column headers) but to the model predicted source (row names). The isolates were assigned to the primary source class with the highest model computed probability of assignment. Balanced accuracy is the average of the sensitivity (true positive rate) and specificity (true negative rate) values for each primary source class.

|  | Broilers | Cattle | Game | Layers | OtherMammals | Pigs | Sheep | Turkey |
| --- | --- | --- | --- | --- | --- | --- | --- | --- |
| Broilers | **14** | 1 | 0 | 0 | 0 | 0 | 0 | 0 |
| Cattle | 0 | **58** | 0 | 0 | 6 | 2 | 8 | 0 |
| Game | 0 | 0 | **14** | 0 | 0 | 0 | 0 | 0 |
| Layers | 0 | 0 | 0 | **5** | 0 | 0 | 0 | 0 |
| OtherMammals | 0 | 0 | 0 | 0 | **35** | 0 | 1 | 0 |
| Pigs | 2 | 1 | 0 | 1 | 3 | **130** | 0 | 0 |
| Sheep | 0 | 2 | 0 | 0 | 1 | 0 | **29** | 0 |
| Turkey | 0 | 0 | 0 | 0 | 0 | 0 | 0 | **9** |
|  |  |  |  |  |  |  |  |  |
| Sensitivity | 0.875 | 0.936 | 1.000 | 0.833 | 0.778 | 0.985 | 0.763 | 1.000 |
| Specificity | 0.997 | 0.939 | 1.000 | 1.000 | 0.996 | 0.963 | 0.989 | 1.000 |
| Balanced  Accuracy | 0.936 | 0.937 | 1.000 | 0.917 | 0.887 | 0.974 | 0.876 | 1.000 |

**Supplementary Table 3B** The tuned RF3 machine learning model confusion matrix for the assignment of 77 test set animal origin *S*. Typhimurium and monophasic *S*. Typhimurium isolates to eight primary source classes. The values along the diagonal (in bold) indicate the number of isolates correctly assigned by the model to their actual primary source class. The values above and below the diagonal indicate the number of isolates incorrectly classed by the model not to their actual primary source class (column headers) but to the model predicted source (row names). The isolates were assigned to the primary source class with the highest model computed probability of assignment. Balanced accuracy is the average of the sensitivity (true positive rate) and specificity (true negative rate) values for each primary source class.

|  | Broilers | Cattle | Game | Layers | OtherMammals | Pigs | Sheep | Turkey |
| --- | --- | --- | --- | --- | --- | --- | --- | --- |
| Broilers | **2** | 0 | 0 | 0 | 0 | 0 | 0 | 0 |
| Cattle | 0 | **13** | 0 | 0 | 1 | 2 | 2 | 0 |
| Game | 0 | 0 | **3** | 0 | 0 | 0 | 0 | 0 |
| Layers | 0 | 0 | 0 | **0** | 0 | 0 | 0 | 0 |
| OtherMammals | 0 | 0 | 0 | 0 | **5** | 0 | 0 | 0 |
| Pigs | 1 | 2 | 0 | 1 | 5 | **30** | 0 | 0 |
| Sheep | 0 | 0 | 0 | 0 | 0 | 0 | **7** | 0 |
| Turkey | 0 | 0 | 0 | 0 | 0 | 1 | 0 | **2** |
|  |  |  |  |  |  |  |  |  |
| Sensitivity | 0.667 | 0.867 | 1.000 | 0.000 | 0.455 | 0.909 | 0.778 | 1.000 |
| Specificity | 1.000 | 0.919 | 1.000 | 1.000 | 1.000 | 0.796 | 1.000 | 0.987 |
| Balanced Accuracy | 0.833 | 0.893 | 1.000 | 0.500 | 0.727 | 0.852 | 0.889 | 0.993 |

**Supplementary Table 4** Comparison of the incorrectly assigned training set animal isolates between the tuned RF1, RF2, and RF3 models.

*Excel table*

**Supplementary Table 5** Comparison of the incorrectly assigned test set animal isolates between the tuned RF1, RF2, and RF3 models.

*Excel table*

**Supplementary Table 6** Comparison of the numbers of human isolates assigned to eight primary source classes by the RF1 and RF1 - no DT104 models.

*Excel table*
